# Individualizing Life Expectancy Estimates for Older Adults Using the Gompertz Law of Human Mortality

**DOI:** 10.1371/journal.pone.0108540

**Published:** 2014-09-29

**Authors:** Sei J. Lee, W. John Boscardin, Katharine A. Kirby, Kenneth E. Covinsky

**Affiliations:** Division of Geriatrics, University of California San Francisco, San Francisco, CA, United States of America; Bielefeld Evangelical Hospital, Germany

## Abstract

**Background:**

Guidelines recommend incorporating life expectancy (LE) into clinical decision-making for preventive interventions such as cancer screening. Previous research focused on mortality risk (e.g. 28% at 4 years) which is more difficult to interpret than LE (e.g. 7.3 years) for both patients and clinicians. Our objective was to utilize the Gompertz Law of Human Mortality which states that mortality risk doubles in a fixed time interval to transform the Lee mortality index into a LE calculator.

**Methods:**

We examined community-dwelling older adults age 50 and over enrolled in the nationally representative 1998 wave of the Health and Retirement Study or HRS (response rate 81%), dividing study respondents into development (n = 11701) and validation (n = 8009) cohorts. In the development cohort, we fit proportional hazards Gompertz survival functions for each of the risk groups defined by the Lee mortality index. We validated our LE estimates by comparing our predicted LE with observed survival in the HRS validation cohort and an external validation cohort from the 2004 wave of the English Longitudinal Study on Ageing or ELSA (n = 7042).

**Results:**

The ELSA cohort had a lower 8-year mortality risk (14%) compared to our HRS development (23%) and validation cohorts (25%). Our model had good discrimination in the validation cohorts (Harrell’s c 0.78 in HRS and 0.80 in the ELSA). Our predicted LE’s were similar to observed survival in the HRS validation cohort without evidence of miscalibration (Hosmer-Lemeshow, p = 0.2 at 8 years). However, our predicted LE’s were longer than observed survival in the ELSA cohort with evidence of miscalibration (Hosmer-Lemeshow, p<0.001 at 8 years) reflecting the lower mortality rate in ELSA.

**Conclusion:**

We transformed a previously validated mortality index into a LE calculator that incorporated patient-level risk factors. Our LE calculator may help clinicians determine which preventive interventions are most appropriate for older US adults.

## Introduction

Patients and families rely on physicians to synthesize a patient’s risk factors into an accurate overall assessment of life expectancy (LE) so that they can plan and prepare for the future [1], [2]. Because preventive interventions such as cancer screening have immediate risks with delayed benefits, patients with limited life expectancy who undergo cancer screening are subject to the immediate harms with little chance that they would survive to benefit. Guidelines now recommend that clinicians estimate patients’ LE and target screening to those patients with an extended life expectancy [3]–[5]. Thus, improving the accuracy of LE prediction could help doctors address common patient and family questions as well as improve the targeting of preventive interventions.

Unfortunately, physician estimates of LE are often inaccurate [2], [6], [7]. Two types of prognostic information are available to help providers more accurately predict LE, but both have important disadvantages which limit their usefulness in routine clinical care. First, numerous mortality prediction indexes have been developed which combine a wide range of patient-level risk factors to predict mortality risk [8]. However, these indexes cannot directly estimate LE. Rather, they provide a risk of mortality at a given point in time in the future [9]. For example, for a 77 year old man with congestive heart failure and difficulty walking several blocks, the Lee index predicts a 4-year mortality risk of 28% [10] and a 10-year mortality risk of 70% [11]. However, because guidelines suggest targeting prevention to those patients with a life expectancy greater than the time-to-benefit for prevention [3], [4], these 4-year and 10-year mortality risk estimates are less helpful than LE in targeting prevention. Further, mortality risk estimates are more difficult to interpret for patients and families. This shortcoming of mortality indexes reflects practical limitations, as it requires following a cohort until most participants have died for a model to predict life expectancy with minimal assumptions.

The second type of prognostic information that clinicians can use to predict LE comes from life tables [12]. Life tables have the advantage of providing a LE estimate; however, unlike mortality risk indexes, life tables provide a single LE prediction for a given age, gender and race/ethnicity. Recent studies have used life table methods while accounting for comorbidities; however, important prognostic factors such as smoking and functional limitations have not been incorporated in LE calculators to date [13], [14]. Because life tables methods ignore factors that are strong predictors of mortality, their face validity to clinicians and patients is low and they have not been widely adopted in clinical practice. What would be most helpful (and what neither mortality indexes nor life tables can do) would be a way to incorporate patient-level clinical risk factors such as smoking status and functional limitations to predict LE.

In 1825, Benjamin Gompertz first observed that mortality risk rises exponentially with age, repeatedly doubling in a fixed time interval [15]. Although mortality rates and doubling times vary across different populations, the Gompertz Law of Human Mortality has been empirically verified over 2 centuries across a wide range of countries and even different species [16]. For example, the 1-year mortality risk for white male Americans in 2000 doubled approximately every 8 years, with 58-year olds having a 1% risk of dying in the next year, 66-year olds having a 2% risk and 74-year olds having a 4% risk [17]. The Gompertz function translates this widely-verified principle of demography into mathematical form and has been successfully used as the statistical basis for survival analysis [18]–[21]. Our objective was to transform a previously validated mortality index with the Gompertz Law of Human Mortality to develop a clinically-useful LE calculator that incorporates patient-level risk factors.

## Methods

### Overview

We started with the assumption that all persons within the same risk group from a mortality index (e.g. all persons with 8 risk points from the Lee index) can be viewed as a distinct subpopulation in the same way as demographically defined groups (e.g. all white male 65 year old Americans). We used the Lee index to group all persons with the same risk score together, effectively defining a series of subpopulations at varying 4-year mortality risk. Then, we fit a Gompertz survival function, with each point score allowed to have a flexible proportional effect on the hazard rate, making the assumption that like all adult populations in the developed world, each subpopulation will experience an exponential rise in mortality risk over time. These fit Gompertz functions allowed us to predict LE (e.g. 17.8 years) rather than mortality risk (e.g. 8% risk at 4 years). To determine the validity of our methods, we compared our predicted LE with the observed LE in an internal HRS validation cohort and an external ELSA validation cohort.

The Lee mortality index identified 12 risk factors that accurately stratified a nationally representative cohort into 15 groups, ranging in 4-year mortality risk from 1% (0 risk point group) to 64% (14+ risk points group) [10]. We fit a Gompertz function with the point score as a categorical predictor affecting the proportional hazard component of the model in the development cohort, allowing us to determine the time to 50% mortality, or median LE, of each risk point group. We validated our Gompertz function predicted LE by comparing to the observed survival experience in our validation cohorts. Using the fit Gompertz functions, we also determined the time to 25% mortality and time to 75% mortality for each risk point group and compared these Gompertz predictions to the observed time to 25% and 75% mortality in our validation cohorts.

### Study Population

Like our previous studies of the Lee index, we examined community-dwelling participants interviewed in 1998 as part of the Health and Retirement Study (HRS), a nationally-representative sample of US adults over age 50 [22]. We divided our cohort into a development cohort (n = 11701) of enrollees living in the East, Midwest and West regions and a validation cohort (n = 8009) of enrollees living in the South region. We used the English Longitudinal Study on Ageing (ELSA) cohort (n = 7042) of English adults over age 50 as a separate, external validation cohort.

### Measures

Our primary predictor was the Lee risk score (range 0–14+), obtained by summing points associated with the 12 items previously found to be strong independent predictors of 4-year mortality (see Table 1) [10]. Our outcome was time to death, which was determined by cross-referencing HRS information with the National Death Index [22].

**Table 1:** Characteristics of Subjects.

Characteristic	Points	HRS DevelopmentCohort (n = 11701)	HRS ValidationCohort (n = 8009)	ELSA ValidationCohort (n = 7042)
Demographics				
Age (y)				
50–59	0	3154 (27)	2328 (29)	2197 (31)
60–64	1	2145 (18)	1547 (19)	1215 (17)
65–69	2	1798 (15)	1188 (15)	1198 (17)
70–74	3	1669 (14)	1017 (13)	971 (14)
75–79	4	1362 (12)	909 (11)	732 (10)
80–84	5	856 (7)	533 (7)	481 (7)
>85	7	715 (6)	487 (6)	248 (4)
Male gender	2	5062 (43)	3493 (44)	3173 (45)
Comorbidities and Behaviors				
Diabetes mellitus	1	1608 (14)	1246 (16)	541 (8)
Cancer	2	1349 (12)	862 (11)	536 (8)
Chronic Lung disease	2	443 (4)	376 (5)	445 (6)
Heart Failure	2	298 (3)	240 (3)	1360 (19)
BMI <25	1	4402 (38)	2981 (37)	1954 (28)
Current smoker	2	1813 (16)	1416 (18)	1019 (14)
Functional measures				
Bathing	2	754 (6)	644 (8)	706 (10)
Managing finances	2	887 (8)	737 (9)	145 (2)
Walking several blocks	2	3167 (27)	2542 (32)	722 (10)
Pushing or pulling heavy objects	1	3329 (28)	2604 (33)	1171 (17)
Point Score				
0		742 (6)	494 (6)	459 (7)
1		1368 (12)	889 (11)	503 (7)
2		1480 (13)	973 (12)	1013 (14)
3		1448 (12)	994 (12)	937 (13)
4		1334 (11)	845 (11)	868 (12)
5		1168 (10)	762 (10)	810 (12)
6		890 (8)	638 (8)	589 (8)
7		762 (7)	501 (6)	517 (7)
8		552 (5)	404 (5)	405 (6)
9		409 (3)	310 (4)	296 (4)
10		324 (3)	233 (3)	202 (3)
11		244 (2)	192 (2)	154 (2)
12		177 (2)	159 (2)	118 (2)
13		315 (3)	260 (3)	67 (1)
14+		488 (4)	355 (4)	104 (1)
**Death in 8 years**		**2676 (23)**	**1986 (25)**	**981 (14)**
**Death in 10 years**		**3408 (29)**	**2527 (32)**	**–**

### Statistical Analysis

We fit a Gompertz survival function in the development cohort to the end of follow up (10 years) and then extrapolated these curves beyond 10 years. The Gompertz model used the standard survival regression parameterization for the hazard function, h_i_(t) = λ_i_exp(γt), where λ_i_ = exp(x_i_β) allows for a proportional hazards specification of the hazard according to the covariates for the ith subject. We used a categorical specification of the point score as the covariates, creating 14 dummy variables and comparing each of the non-zero point scores to zero points. The fitted Gompertz model allowed us to determine the time to 25%, 50% and 75% mortality for each of the risk point groups. Bootstrap methods (1000 replications) were used to determine the 95% confidence intervals around our time to mortality estimates.

In addition to 95% confidence intervals for the median survival, we report 50% prediction intervals. Confidence intervals convey the potential range of the (unknown) true median survival consistent with the sampled data. Thus, a predicted LE with a 95% CI of 10 to 20 years suggests 95% confidence that the true median survival is between 10 to 20 years. Prediction intervals convey the range of potential values for an individual patient’s experience, incorporating both the uncertainty of our estimates due to population sampling as well as the variability between individuals. Thus, a predicted LE with a 50% prediction interval of 10 and 20 years suggests that an individual patient has a 50% probability of surviving 10–20 years.

To validate our assumptions, we first compared our fit Gompertz model with Weibull, Gamma, Log-logistic, Log-normal and exponential models using Akaike and Bayesian information criteria. Second, we compared our fit Gompertz model with observed Kaplan-Meier (KM) survival curves for the HRS development cohort, HRS validation cohort and ELSA validation cohort. Model calibration was assessed using graphical techniques (examining how well the KM curve for a given point score tracks the predicted survival curve) and the Hosmer-Lemeshow test. [23]. Because HRS follow-up was limited to slightly over 10 years and ELSA follow-up was limited to 8 years, our validations are limited to these time frames. However, we also present Gompertz-predicted LE’s beyond 10 years to guide clinicians in predicting extended LE. Finally, we calculated Harrell’s c-statistic as our global measure of model discrimination. The Committee on Human Research at the University of California, San Francisco approved this study. STATA 12.1 (College Station, TX) and SAS 9.2 (Cary, NC) were used for statistical analysis. De-identified HRS and ELSA data is available without cost to registered users. Additional details on obtaining HRS and ELSA data is available at http://hrsonline.isr.umich.edu/ and http://www.elsa-project.ac.uk/.

## Results

Fifty-five percent of the HRS development cohort was over 65 and 43% was male (Table 1). Fifty-two percent of the HRS validation cohort was over 65 and 44% was male. Fifty-two percent of the ELSA validation cohort was over 65 and 45% was male. The HRS development and validation cohorts were similar with 7% having 13 or 14+ points. In contrast, the ELSA cohort was healthier, with only 2% having 13 or 14+ points. ELSA cohort had a lower 8 year mortality rate at 14%, compared the the HRS development cohort (23%) and the HRS validation cohort (25%).

**Figure 1:**
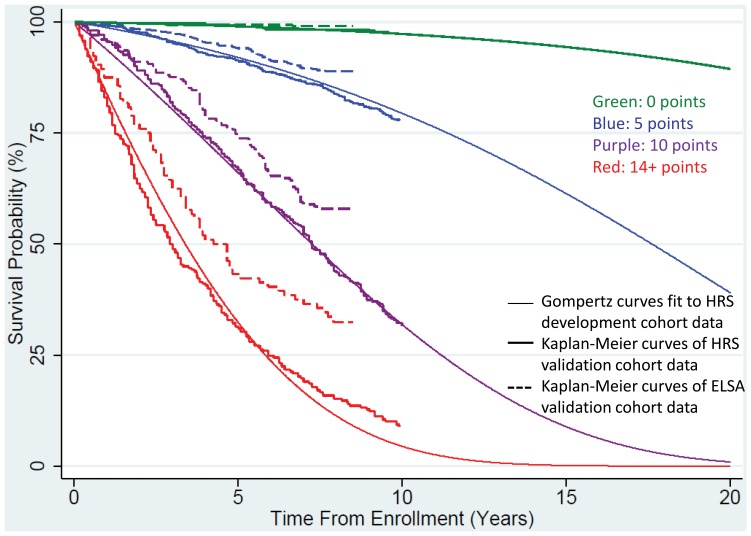
Life expectancy estimations using survival curve models and validation cohorts.

We performed serial diagnostic tests of our prediction model. First, compared to Weibull, Gamma, Log-Logistic, Log-Normal and Exponential models, we found that our Gompertz model yielded by far the best fit to the HRS development cohort data with the lowest AIC and BIC (Gompertz: AIC = 17938.6, BIC = 18056.4; next best fit was Weibull: AIC = 18030.3, BIC = 18154.2). Second, we compared the Gompertz model survival curves with observed KM survival curves for the HRS development (not shown), HRS validation and ELSA validation cohorts (see Figure 1). We found excellent agreement between the model prediction curves, HRS development and HRS validation cohort KM curves. However, we found that the ELSA validation cohort was less likely to die throughout follow-up, reflecting their lower mortality rate. The Hosmer-Lemeshow test reflected these results, with no evidence of miscalibration for the HRS development and validation cohorts (p = 0.4, 0.9 and 0.9 for HRS development cohort at 5, 8 and 10 years; p = 0.2, 0.2 and 0.2 for HRS validation cohort at 5, 8 and 10 years). However, for the ELSA validations cohort, the Hosmer-Lemeshow test showed evidence of miscalibration (p<0.001 and both 5 and 8 years).

The figure shows that the Gompertz-predicted survival curve, the observed KM survival curve from the HRS validation cohort and the observed KM survival curve from the ELSA validation cohort for 4 selected risk point subpopulations (0 points, 5 points, 10 points and 14+ points). The lower mortality rates in the ELSA cohort is reflected in figure 1 with higher survival rates at each time point. HRS follow-up was limited to slightly over 10 years andELSA follow-up was limited to 8 years resulting in KM curves being truncated at 10 and 8 years, respectively.The results for all other risk point subpopulations were similar to the 4 risk points shown.

Table 2 shows our validation comparing our Gompertz-predicted LE to the observed survival in the HRS development, HRS validation and ELSA validation cohorts. For example, for participants with 11 risk points, the Gompertz-predicted LE is 5.9 years (95% CI: 5.3, 6.4), while the HRS development cohort observed median survival is 5.4 years, the HRS validation cohort observed median survival is 5.0 years and the ELSA validation cohort observed median survival is 7.4 years. The Harrell’s c-statistic was 0.790 in the HRS development cohort,0.779 in the HRS validation cohort and 0.801 in the ELSA validation cohort. For the low risk point scores (0–7 points), less than 50% had died in both the development and validation cohorts at the end of HRS follow-up (10 years); thus, we report the observed survival in both the development and validation cohorts for 0–7 points as >10 years. Similarly, our ELSA follow-up was 8 years, thus we report observed survival for 0–10 points as >8 years.

**Table 2:** Validation of Gompertz-Predicted Median Life Expectancy by Risk Points.

	HRS Development Cohort		HRS Validation Cohort	ELSA Validation Cohort
PointScore	Gompertz-Predicted Median Life Expectancy,in years (95% CI), [50% PI]	Observed MedianSurvival, in years	Observed MedianSurvival, in years	Observed MedianSurvival, in years
**0**	35.4 (31.0–39.8) [28.5–42.5]	>10*	>10*	>8*
**1**	33.1 (29.9–36.2) [23.7–37.5]	>10*	>10*	>8*
**2**	30.1 (28.1–32.0) [22.0–35.6]	>10*	>10*	>8*
**3**	23.7 (22.4–24.9) [16.5–29.6]	>10*	>10*	>8*
**4**	21.1 (20.0–22.1) [14.1–26.7]	>10*	>10*	>8*
**5**	17.7 (16.8–18.5) [11.2–23.0]	>10*	>10*	>8*
**6**	14.3 (13.7–15.0) [8.4–19.2]	>10*	>10*	>8*
**7**	12.6 (12.0–13.2) [7.0–17.2]	>10*	>10*	>8*
**8**	10.3 (9.7–10.9) [5.6–14.9]	9.9	10.0	>8*
**9**	8.8 (8.2–9.3) [4.8–13.3]	8.3	8.9	>8*
**10**	7.3 (6.7–7.8) [3.8–11.4]	6.8	7.2	>8*
**11**	5.9 (5.3–6.4) [2.9–9.3]	5.4	5.0	7.4
**12**	5.3 (4.7–5.8) [2.6–8.8]	4.6	5.1	6.7
**13**	4.8 (4.1–5.4) [2.2–7.6]	4.0	3.8	6.5
**≥14**	3.4 (3.0–3.8) [1.7–6.2]	3.1	2.9	4.2
**Harrell’s c-statistic**		0.790	0.779	0.801

*Follow-up for HRS is limited to 10 years while the follow-up for ELSA is limited to 8 years.

CI is confidence interval.

PI is prediction interval.

Table 3 shows the Gompertz-predicted time to 25% mortality, median LE and time to 75% mortality compared to the observed time to 25% mortality, median survival and time to 75% mortality for each of the 15 risk point groups. For example, for a patient with 11 risk points, their predicted time to 25% mortality risk is 2.9 years (95% CI: 2.6, 3.2), their median LE is 5.9 years (95% CI: 5.3, 6.4) and their time to 75% mortality risk is 9.4 years (95% CI: 8.6, 10.1). The HRS validation cohort observed time to 25% mortality is 2.7 years, the observed median survival is 5.0 years and the observed time to 75% survival is 9.1 years. The ELSA validation cohort observed time to 25% mortality is 3.8 years and the observed time to median survival is 7.4 years.

**Table 3:** Predicted and Observed Median Life Expectancy, Time to 25% Mortality and Time to 75% Mortality by Risk Points.

	Time to 25% Mortality in years			Median Life Expectancy in years(Time to 50% Mortality)			Time to 75% Mortalityin years		
Points	Predicted from HRSDevelopmentCohort (95% CI)	Observed in HRSValidation Cohort	Observed in ELSAValidation Cohort	PredictedFrom HRSDevelopmentCohort (95% CI)	Observed in HRSValidation Cohort	Observed in ELSAValidation Cohort	PredictedFrom HRSDevelopmentCohort (95% CI)	Observed in HRSValidation Cohort	Observed in ELSAValidation Cohort
**0**	27.8 (23.5–32.1)	>10	>8	35.4 (31.0–39.8)	>10	>8	41.5 (36.9–46.0)	>10	>8
**1**	25.5 (22.6–28.4)	>10	>8	33.1 (29.9–36.2)	>10	>8	39.1 (35.8–42.4)	>10	>8
**2**	22.7 (20.9–24.4)	>10	>8	30.1 (28.1–32.0)	>10	>8	36.1 (33.8–38.3)	>10	>8
**3**	16.7 (15.7–17.6)	>10	>8	23.7 (22.4–24.9)	>10	>8	29.5 (27.9–31.0)	>10	>8
**4**	14.4 (13.5–15.2)	>10	>8	21.1 (20.0–22.1)	>10	>8	26.8 (25.5–28.1)	>10	>8
**5**	11.4 (10.7–12.1)	>10	>8	17.7 (16.8–18.5)	>10	>8	23.2 (22.1–24.2)	>10	>8
**6**	8.7 (8.2–9.2)	8.3	>8	14.3 (13.7–15.0)	>10	>8	19.6 (18.7–20.4)	>10	>8
**7**	7.4 (6.9–7.9)	6.1	>8	12.6 (12.0–13.2)	>10	>8	17.6 (16.8–18.4)	>10	>8
**8**	5.8 (5.4–6.1)	5.6	6.3	10.3 (9.7–10.9)	10.0	>8	14.9 (14.2–15.6)	>10	>8
**9**	4.7 (4.3–5.1)	5.0	6.1	8.8 (8.2–9.3)	8.9	>8	13.1 (12.4–13.7)	>10	>8
**10**	3.8 (3.4–4.1)	3.9	4.9	7.3 (6.7–7.8)	7.2	>8	11.2 (10.5–11.9)	>10	>8
**11**	2.9 (2.6–3.2)	2.7	3.8	5.9 (5.3–6.4)	5.0	7.4	9.4 (8.6–10.1)	9.1	>8
**12**	2.6 (2.2–3.0)	2.6	3.9	5.3 (4.7–5.8)	5.1	6.7	8.6 (7.8–9.3)	9.2	>8
**13**	2.3 (1.9–2.7)	2.1	3.4	4.8 (4.1–5.4)	3.8	6.5	7.8 (6.9–8.7)	6.4	>8
**14+**	1.6 (1.4–1.8)	1.3	2.3	3.4 (3.0–3.8)	2.9	4.2	5.9 (5.2–6.5)	5.9	>8

All predicted values are derived from the Gompertz function fit to HRS development cohort data.

## Discussion

### Overview

We developed and validated a novel methodology to estimate LE using the Lee risk point score, a summary measure combining 12 clinical, patient-level risk factors. We applied the widely verified Gompertz Law of Human Mortality, which allowed us to estimate LE using risk factors such as age, smoking, comorbidities and functional limitations. By accounting for patient-level risk factors, our LE predictions improve upon the LE predictions from life tables, which present a single LE estimates for all persons of a given age, gender and race/ethnicity. Further, our LE estimates (e.g. 6 years) will likely be more intuitive and useful for clinicians, patients and families than mortality indexes which provide a risk of mortality at a fixed point in time (e.g. 30% mortality risk at 4-years) [9].

Our external validation results suggest that our LE predictions are most appropriate for US populations. We found that the ELSA cohort had a lower mortality rate overall and for each point score, had a lower mortality rate throughout the follow-up period compared to HRS cohorts. However, the Harrell’s c-statistic in the ELSA validation cohort was comparable to the Harrell’s c-statistic in HRS, suggesting that the same factors that predicted mortality in HRS were important predictors in ELSA. Previous studies have found that differences in validation populations can lead to miscalibration without decreasing discrimination [24]. Further, miscalibrated prediction models with good discrimination can often be re-calibrated in the new population of interest to yield accurate predictions, suggesting that with re-calibration with ELSA survival data, our index may be able to accurately predict life expectancy for older English adults [25], [26].

### Clinical and Research Relevance

Our results represent an important advance for the field of prognosis for 2 reasons. First, our methodology could be followed for other previously developed mortality indexes to determine LE’s. For example, many mortality prediction indexes have been developed to predict x-year mortality risk for general populations [8] as well as for patients with a dominant condition such as breast cancer, heart failure or obstructive lung disease [27]–[29]. For many of these indexes, our methodology could be used to determine a LE rather than a mortality risk at a given point in time, which may be easier to interpret for clinicians, patients and families.

Second, our work could simplify the targeting of preventive interventions. One barrier to the widespread use of prognosis in clinical decision making has been the need to use different prognostic indexes for different clinical decisions [9]. For example, to determine whether intensive blood pressure control is indicated, a 2-year mortality index may be ideal [8]. However, to determine whether prostate cancer screening is indicated, a 10–15 year mortality index is needed [4]. The practical challenges of finding and completing different mortality indexes for different clinical decisions was a substantial barrier to the routine use of prognosis in clinical care. Since our results suggest that our LE calculator is accurate over a wide range of LE’s, clinicians can focus on obtaining data for 1 LE calculator and use the results to guide decisions on a wide range of preventive interventions.

One specific setting where calculating LE may be especially helpful is the Medicare Annual Wellness Visit. With LE information for an individual patient, a variety of preventive interventions with varying lagtimes to benefit can be considered, with the clinician recommending those interventions with lagtimes to benefit less than the patient’s LE. For example, for an older patient with a total of 12 points and an estimated LE of 5.1 years, colorectal cancer screening may be more likely to harm than help since the lagtime to mortality benefit for colorectal cancer screening has been estimated to be 10.3 years [30]. Conversely, for the same patient, continued blood pressure control and lipid control is probably indicated since the lagtime to benefit for these conditions is estimated to be 2–3 years [8].

### Our Study in Context of Previous Research

Although previous authors have noted that a LE calculator that accounts for clinical risk factors could be useful [9], there has been surprisingly little published research. Previous work has focused on life tables, which are often stratified by race/ethnicity, gender and age but generally do account for clinical risk factors such as smoking and functional limitations [12]. Tan and colleagues used administrative data (demographics and comorbidities) to develop indexes for 1, 5, 7 and 10 years [14]. Cho and colleagues used similar data to identify patients whose mortality risk is similar to chronologically older (or younger) patients and then used life tables to estimate LE [13]. However, neither study incorporated risk factors such as functional limitations or smoking which are strong predictors of mortality in older adults. Keeler and colleagues used the Established Populations for Epidemiologic Studies of the Elderly (EPESE) cohort to extend life table methodologies and determine LE’s for older persons based on their age, gender and functional status (independent, mobility disabled and activity of daily living disabled) [31]. Our methodology builds on this previous work and allows additional clinical risk factors beyond a 3-level functional status variable to be incorporated into LE calculations.

### Strengths and Weaknesses

Our results should be interpreted in light of our project’s strengths and limitations. Strengths of our methodology include our ability to incorporate many clinical risk factors into LE estimates as well as the good agreement between the predicted and observed mortality experience up to 10 years. Limitations include the following.

First, we could not validate our LE estimates beyond 10 years. However, the good agreement between our predicted LE and validation cohort observed LE <10 years suggests that our predicted LE’s beyond 10 years may be accurate as well. Further, assumptions are unavoidable when making LE predictions; LE estimates from life tables which inform planning and policy debates predict LE by assuming that the current mortality experience will remain unchanged many decades into the future. The wide range of empiric validations of the Gompertz Law of Human Mortality suggests that the assumptions that underpin our LE predictions are reasonable.

Second, like mortality indexes, our LE estimates should supplement clinical judgment rather than replace it. Clinicians must judge whether an individual patient is similar (or different) from the research participants that helped develop the index; the more different an individual patient is from the research participants, the less likely that our LE estimates will be accurate for that patient. The Lee index was developed and validated in a nationally representative, community-dwelling sample of older Americans and the evidence of miscalibration in the ELSA validation cohortsuggests that our prediction model should be re-calibrated for older adults in other countries.

Third, the Gompertz Law and function assumes that mortality risk will double in a fixed time interval. This assumption may be invalid if near-term mortality is governed by different processes than long-term mortality. Since the Lee index was known to accurately predict both 4-year and 10-year mortality [10], [11], we had evidence to suggest that our index was able to capture the important factors that affect mortality through 10 years. In contrast, for indexes which predict mortality after hospitalization, near-term mortality is likely governed by the disease processes which led to hospitalization, whereas long-term mortality is likely governed by the same factors as the general population. Thus, fitting a single Gompertz function onto post-hospitalization mortality indexes may not be appropriate.

Fourth, single-number survival predictions for individual patients may provide a false sense of precision given the tremendous uncertainty in predictions for patients (as opposed to populations) [32]. Thus, we encourage both clinicians and patients to focus on the prediction interval to obtain a realistic range of most likely survival experiences for an individual patient.

## Conclusions and Future Directions

We transformed a previously developed mortality index into a LE calculator and validated our predictions through 10 years. The external validation with the ELSA cohort of older English adults showed excellent discrimination but miscalibration, suggesting that our models should be re-calibrated before use in non-US populations. Further, our methodology may be useful to transform other previously developed mortality indexes into life expectancy calculators.
